# An infant formula with large, milk phospholipid–coated lipid droplets containing a mixture of dairy and vegetable lipids supports adequate growth and is well tolerated in healthy, term infants

**DOI:** 10.1093/ajcn/nqy322

**Published:** 2019-02-22

**Authors:** Laura M Breij, Marieke Abrahamse-Berkeveld, Yvan Vandenplas, Sabine N J Jespers, Amerik C de Mol, Poh Choo Khoo, Masendu Kalenga, Stefaan Peeters, Ron H T van Beek, Obbe F Norbruis, Stefanie Schoen, Dennis Acton, Anita C S Hokken-Koelega, Mercurius Study Group

**Affiliations:** 1 Erasmus University Medical Centre/Sophia Children's Hospital, Rotterdam, The Netherlands; 2 Nutricia Research, Utrecht, The Netherlands; 3 Universitair Ziekenhuis, Brussel, Belgium; 4 Clinique et Maternité Sainte-Elisabeth, Namur, Belgium; 5 Albert Schweitzer Ziekenhuis, Dordrecht, The Netherlands; 6 KK Women's and Children's Hospital, Singapore; 7 Centre Hospitalier Régional de la Citadelle, Liège, Belgium; 8 Algemeen Stedelijk Ziekenhuis, Aalst, Belgium; 9 Amphia Ziekenhuis, Breda, The Netherlands; 10 Isala Zwolle, Zwolle, The Netherlands

**Keywords:** Safety, Infant growth, Lipid droplet structure, stool characteristics, dairy lipids

## Abstract

**Background:**

Lipid droplets in human milk have a mode diameter of ∼4 μm and are surrounded by a native phospholipid-rich membrane. Current infant milk formulas (IMFs) contain small lipid droplets (mode diameter ∼0.5 μm) primarily coated by proteins. A concept IMF was developed mimicking more closely the structure and composition of human milk lipid droplets.

**Objectives:**

This randomized, controlled, double-blind equivalence trial evaluates the safety and tolerance of a concept IMF with large, milk phospholipid–coated lipid droplets (mode diameter 3–5 μm) containing vegetable and dairy lipids in healthy, term infants.

**Methods:**

Fully formula-fed infants were enrolled up to 35 d of age and randomly assigned to 1 of 2 formulas until 17 wk of age: *1*) Control IMF with small lipid droplets containing vegetable oils (*n* = 108); or *2*) Concept IMF with large, milk phospholipid–coated lipid droplets comprised of 48% dairy lipids (*n* = 115). A group of 88 breastfed infants served as reference. Primary outcome was daily weight gain during intervention. Additionally, number and type of adverse events, growth, and tolerance parameters were monitored.

**Results:**

Equivalence of daily weight gain was demonstrated (Concept compared with Control IMF: −1.37 g/d; 90% CI: −2.71, −0.02; equivalence margin ± 3 g/d). No relevant group differences were observed in growth, tolerance and number, severity, or relatedness of adverse events. We did observe a higher prevalence of watery stools in the Concept than in the Control IMF group between 5 and 12 wk of age (*P* < 0.001), closer to the stool characteristics observed in the breastfed group.

**Conclusions:**

An infant formula with large, milk phospholipid–coated lipid droplets containing dairy lipids is safe, well tolerated, and supports an adequate growth in healthy infants. This trial was registered in the Dutch Trial Register (www.trialregister.nl) as NTR3683.

## Introduction

Exclusive human milk is the preferred feeding for infants and provides a complete supply of nutrients to support growth and development in early life. Because breastfeeding may not always be possible, breast milk substitutes should aim to provide nutritional and functional properties as close as possible to those of human milk.

Lipids are crucial in fulfilling nutritional needs: the lipid fraction of milk provides almost half of the caloric intake that infants need (1). The lipid droplets in human milk have a volume-based mode diameter of 3–5 μm and are enveloped by a 3-layered membrane mainly consisting of phospholipids, membrane-specific proteins, and cholesterol (2–4). The lipid droplets in most infant milk formulas (IMFs) have a volume-based mode diameter of ∼0.5 µm, contain vegetable oils, and have proteins as their main emulsifier. Over the past decades, substantial improvements in the nutritional (lipid) quality of IMFs have been realized (5). The addition of structured lipids or dairy lipids to increase the sn-2 palmitic acid to levels closer to human milk has previously been shown to reduce calcium soaps, soften stools, and improve fatty acid absorption (6–11). More recently, an adapted production process resulted in an IMF containing larger lipid droplets (3–5 μm) with a thin interface of milk phospholipids, other polar lipids, (glyco)proteins, and cholesterol (12). This concept IMF was shown to alter in vitro lipid digestion kinetics (13) and the postprandial lipid response in adult men (14), and it prevented excessive fat accumulation and adverse metabolic outcomes in a murine nutritional programming model (15, 16) as well as improved specific cognitive behaviors (17) compared with standard IMFs. Hence, introducing large, phospholipid-coated lipid droplets partly comprised of dairy lipids (containing sn-2 palmitic acid) might bring the physiologic properties of IMFs closer to those of human milk. However, stringent clinical evaluation of the nutritional adequacy and safety of this approach is first required (18, 19).

The primary objective of the current study was to evaluate growth of infants, defined as daily weight gain, fed with a concept IMF comprising large, milk phospholipid–coated lipid droplets containing a mixture of dairy and vegetable lipids. Secondary objectives included evaluation of other anthropometric measures, tolerance, stool characteristics, fat-soluble vitamins in plasma, and adverse events (AEs).

## Methods

### Participating centers

This study was conducted in a total of 17 study centers in 4 countries including the Netherlands (6 centers: Erasmus University Medical Centre/Sophia Children's Hospital, Rotterdam; Albert Schweitzer Ziekenhuis, Dordrecht; Amphia Ziekenhuis, Breda; Isala Zwolle, Zwolle; Medisch Spectrum Twente, Enschede; Jeroen Bosch Ziekenhuis,  Hertogenbosch), Belgium (7 centers: Algemeen Stedelijk Ziekenhuis, Aalst; Universitair Ziekenhuis, Brussels; AZ Sint Vincentius, Antwerpen; Clinique et Maternité Sainte-Elisabeth, Namur; Centre Hospitalier Régional de la Citadelle, Liège; Private Practice Dr Franckx, Mollem-Asse; Heilig Hart Ziekenhuis, Roeselare), France (3 centers: CHU de Nantes, Nantes; CHU Angers Unité de Néonatologie, Angers; Hopital Nord-Ouest, Villefranche-sur-Sâone) and Singapore (KK Women's and Children's Hospital, Singapore). All participating centers obtained the approval of their independent local ethics review board. The study was conducted according to International Council for Harmonisation of Technical Requirements for Pharmaceuticals for Human Use (ICH)-good clinical practice (GCP) principles and in compliance with the principles of the Declaration of Helsinki and with the local laws and regulations of the countries where the study was performed. The study was registered in the Dutch Trial Register as NTR3683.

### Subjects and study design

Healthy term infants, with a gestational age of between 37 and 42 wk, postnatal age ≤35 d, a birth weight between the 10th and 90th percentiles according to the Dutch Growth Charts (20), a head circumference within normal range for age and sex [within 2 SDs according to WHO Growth Standard (21)], and either fully formula-fed or fully breastfed were eligible for participation. The recruitment period was extended in order to enroll additional infants with postnatal age ≤14 d in order to fulfil the requirements of the FDA safety assessment guidance (18). Exclusion criteria were defined as illnesses that could interfere with the study, special dietary needs, diagnosed maternal hepatitis B or HIV, participation in any other study, or investigator's uncertainty about the ability of the parents to comply with the protocol requirements. Written informed consent was obtained from all parents/guardians before enrollment. The study was designed as a randomized, double-blind, controlled, prospective, multicountry, (growth) equivalence trial. Formulas were coded by the sponsor (only known by the clinical studies supplies manager) as letter codes A, B, C, and D; both the investigators and the infants’ parents were blinded to the formulas. The randomization sequence was generated based on region (Europe/Asia), sex (male/female), and infants’ age at randomization (≤14 d/˃14 d) as strata (PLAN procedure in SAS statistical software; Enterprise Guide version 4.3) by a statistician from Nutricia Research who had no further involvement in the conduct of the study. The generated randomization sequence was uploaded in the eCRF (electronic case report form) and, after enrollment and input of subject data, formula-fed infants were randomly assigned by the investigator to receive 1 of 2 formulas. Inclusion of twins was allowed with one twin being randomly assigned and the second twin assigned to the same product. Breastfed infants served as a reference group and were eligible if the mother intended to (fully) breastfeed for at least 13 wk and all other inclusion criteria were met. During the study, infants were fully formula-fed or fully breastfed; only use of water, tea, rehydration solutions, drops or syrups (vitamins, minerals, medicines) was allowed.

### Study products

The intervention formulas were isocaloric (66 kcal/100 ml), contained similar amounts of protein (1.3 g/100 mL), lipids (3.4 g/100 mL), and scGOS/lcFOS prebiotic mixture, a specific prebiotic mixture consisting of short-chain galacto-oligosaccharides and long-chain fructo-oligosaccharides (9:1, 0.8 g/100 mL), and were manufactured per good manufacturing practices (ISO 22000) and compliant with Directive 2006/141/EC (**Table 1**). The key differences between the study IMFs were the following: *1*) the size of their lipid droplets, *2*) the coating of their lipid droplets, and *3*) the origin of their lipid sources. The Control IMF was a vegetable oil–based standard IMF containing lipid droplets with a volume-based mode diameter of 0.5 μm and proteins as main emulsifiers. The Concept IMF contained a mixture of vegetable (52%) and dairy lipids (48%), including milk phospholipids, introducing a 3-fold increase of sn-2 palmitic acid compared with the Control IMF (36% compared with 12% of total palmitic acid). The lipid droplets in the Concept IMF had a volume-based mode diameter of 3–5 μm and an interface predominantly composed of milk phospholipids following an adapted production process (12) (Nuturis, patent EP2825062A1; Nutricia Research).

**TABLE 1:** Composition of the intervention products^1^

Per 100 mL	Control IMF	Concept IMF
Energy, kcal	66	66
Fat, g	3.4	3.4
Vegetable oil, g	3.3	1.7
Dairy lipids, g	0.1	1.6
Saturates, g	1.5	1.4
Monounsaturates, g	1.3	1.2
Polyunsaturates, g	0.6	0.6
Linoleic acid, mg	447	447
α-linolenic acid, mg	82	83
Arachidonic acid, mg	11	12
Eicosapentaenoic acid, mg	1.4	1.8
Docosahexaenoic acid, mg	6.4	6.6
Palmitic acid, mg	580	566
sn-2 Palmitic acid, mg	67	202
Milk phospholipids, mg	—	55
Soy phospholipids, mg	4.5	—
Protein, g	1.3	1.3
Whey protein, g	0.8	0.8
Casein, g	0.5	0.5
Carbohydrates, g	7.3	7.3
scGOS/lcFOS (9:1), g	0.8	0.8
Vitamins		
Vitamin A, μg RE	50	61
Vitamin E, mg α-TE	1.1	1.1
α-Tocopherol, mg	1.3	1.3

1 IMF, infant milk formula; RE, retinol equivalent; scGOS/lcFOS (9:1), a specific prebiotic mixture consisting of short-chain galacto-oligosaccharides and long-chain fructo-oligosaccharides in a ratio of 9:1; α-TE, α-tocopherol equivalent.

### Measurements

The primary outcome measure of the study was the daily weight gain (g/d) from enrollment until 17 wk of age. Secondary outcome measures included length, head circumference, formula intake, tolerance parameters, plasma parameters, and AEs.

Infants had an enrollment (baseline) visit ≤35 d of age, followed by visits at 5, 8, 13, and 17 wk of age. Demographic information and infant characteristics were collected at the enrollment visit. If the baseline was conducted within 2 wk before the visit at 5 wk, parents were only invited again to the study center when their infants were aged ≥8 wk.

Anthropometric parameters were measured at enrollment (baseline) and each visit thereafter. Infants were weighed twice naked, on calibrated electronic scales. Supine length of infants was measured twice with the use of a standard measuring board. A nonstretchable measuring tape was used to measure head circumference in duplicate. If the anthropometric measures deviated substantially (>100 g for weight and >5 mm for length and head circumference), a third measurement was obtained and the measures closest together were averaged as the outcome measurement.

Daily study product intake, gastrointestinal symptoms, and stool characteristics were recorded by the parents in diaries spanning the 7-d period preceding each visit. The severity of gastrointestinal symptoms (cramps, diaper rash, regurgitation, and vomiting) was recorded once a day on a 4-point scale (1 = absent, 2 = mild, 3 = moderate, and 4 = severe). Stool consistency was scored for each stool passed, on a 4-point scale based on the use of pictures (1 = watery, 2 = soft, 3 = formed, 4 = hard) according to the “Amsterdam” stool form scale (22).

During the study, AEs, including serious (S) AEs, were documented by the investigators at each visit, including onset, duration, severity and seriousness, relationship with the study product, any actions that were taken, and the outcomes. In addition, 4 telephone calls (at 6, 11, 15, and 19 wk of age) were conducted to record changes in or any new (S)AEs, use or change of medication and nutritional supplements, and compliance with the study protocol. (S)AEs were followed-up by the investigator until they had abated or until a stable situation had been reached.

At 13 wk of age, a blood sample was drawn via heel prick (Microtainer tubes, Becton Dickinson) from infants whose parents gave additional consent for this procedure. The (heparin) plasma was stored at −80°C until shipment to Nutricia Research. Fat-soluble vitamin levels were determined to evaluate a potential effect of the altered lipid droplet characteristics in the Concept IMF on their bioavailability. Retinol and α-tocopherol plasma content was determined by HPLC, through the use of UV-absorbance for detection of retinol and fluorometric properties for detection of α-tocopherol, by comparing with standard solutions.

### Statistics

The primary outcome of the study was the daily weight gain (g/d) from enrollment until 17 wk of age. Equivalence was demonstrated when the 2-sided 90% CI of the difference in means of daily weight gain lay within the predefined −0.5 SD to +0.5 SD equivalence margins, with a minimum of 3 g/d (18) and a maximum value of 5 g/d. The required sample size for two 1-sided statistical tests based on an SD of 6.0 g/d, α = 0.05, a power = 0.80, and assuming no daily weight gain differences between the 2 intervention groups, was 70 infants per intervention group. Assuming a drop-out rate of 20%, a total of 176 infants (88 per group) needed to be enrolled. The protocol was amended to fulfil the requirements of the FDA for safety studies (i.e., inclusion of enough infants ≤14 d of age at the time of full formula feeding). The initial sample size calculation for this group of young infants was based on the same a priori estimates as described above except an SD of difference equal to 5.5 g/d was used. Assuming a drop-out rate of 20%, a total of 150 infants (75 per group) needed to be enrolled. An interim analysis including a re-estimation of the sample size was performed and evaluated by an independent Data Monitoring Committee. The Data Monitoring Committee advised increasing the sample size to 152 infants to meet the requirements for the group of young infants (≤14 d of age).

Equivalence analyses for weight gain, length gain, and head circumference gain were performed with the use of the parametric curve mixed model (PC) which describes the development of growth parameters over time by a second-order polynomial curve, with the stratification factors as a fixed effect and each subject's intercept and slope as random effects. Sensitivity analyses to evaluate robustness of results were performed with the use of the general linear model (GLM, ANCOVA) on the 17 wk of age measurement with the baseline measurement as covariate and a mixed model with the use of the age variable as a categoric variable. Additionally, WHO growth standard *z* scores (21) were analyzed with the use of a mixed model having age as a categoric variable (i.e., repeated measures for visit with unstructured covariance matrix) and with stratification factor as fixed terms and the interactions as sex by visit and group by visit. In any other analyses, 2-sample *t* tests (TT) were used for continuous data and Mann-Whitney *U* tests (MW) were used if the assumption normality was violated. Categoric response parameters were analyzed by the use of chi-square tests (C; Fisher's exact tests in case sparse cells occurred; FE). In the intention-to-treat (ITT) analysis, the data of all infants randomly assigned to study product (or being breastfed) were used. In the per-protocol (PP) analysis, eligibility of data was assessed on the visit level, and data of breastfed and formula-fed infants were excluded when other formulas or solid foods were consumed for >3 d before any visit until 13 and 17 wk of age, respectively. For drop-outs, the available data until drop-out were considered in the statistical analyses; no data imputation was performed.

Evaluation of tolerance outcomes only included subject's diary data when at least 3 informative days were available (per visit period). Stool consistency was evaluated by calculating for each subject, at each visit, with the mean stool consistency rounded to an integer number after which the subject was placed into the corresponding stool consistency category. The incidence of diarrhea and constipation was derived from the stool frequency and consistency score based on the individual diary information of the period prior to a visit. Derived from the WHO definition, diarrhea was defined as present when a subject passed “3 or more watery stools on at least 1 day” for at least 1 d. Constipation was defined as present if a subject passed “at most 2 stools per week, all of hard consistency” [adapted Rome III criteria (23)]. For the evaluation of the gastrointestinal (GI) symptom severity score, for each subject, at each visit, the mean GI symptom severity score was calculated and rounded to an integer number after which the subject was placed into the corresponding GI symptom severity score category. The frequencies of the stool consistency scores, diarrhea, and constipation, as well as of the GI symptom severity scores, were compared between the formula groups at each visit (with the use of the relevant statistical tests as mentioned above). All statistical analyses were performed with SAS (SAS Enterprise Guide 4.3 or higher) for Windows (SAS Institute Inc.). For each *P* value reported, the type of statistical analysis is indicated with its abbreviation.

## Results

### Subject characteristics

**FIGURE 1:**
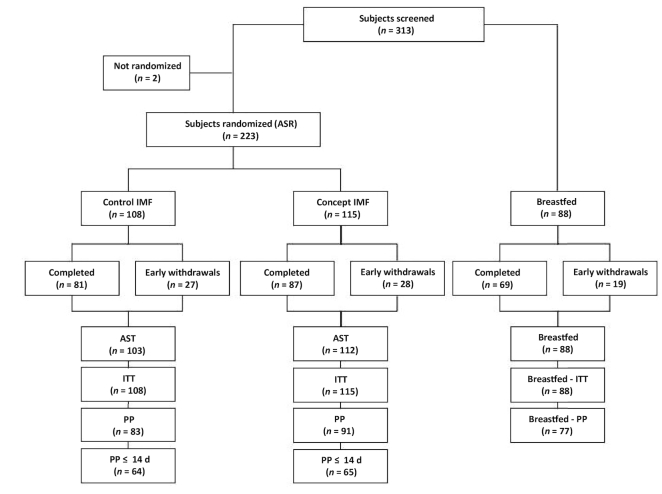
Flow chart of progression of infants during the study. Not randomized = infants not eligible for participation; ASR, all subjects randomized; AST, all subjects treated; IMF, infant milk formulation; ITT, intention-to-treat population; PP, per-protocol population; PP ≤ 14 d = fully formula-fed infants enrolled before 14 d of age and protocol compliant. Protocol compliance was based on visit level, explaining the higher number in the PP compared with the Completers population within all 3 study groups.

From October 2012 to January 2014, 313 subjects were screened for eligibility, 223 subjects were randomized to one of the infant formulas; 88 infants were included in the breastfed reference group (**Figure 1**), 2 subjects did not meet the eligibility criteria. Eight randomly assigned infants did not consume any study product and were excluded from the all subjects treated (AST) population. A total of 168 randomly assigned subjects completed the intervention period, of which 81 subjects were in the Control IMF and 87 subjects in the Concept IMF group, resulting in a drop-out rate of 25%. A total of 69 breastfed infants completed the study up to 17 wk of age (22% drop-out). The drop-out rate (Control 25% compared with Concept 24%, *P* = 0.910 C) and reasons for early termination were similar between both formula groups (*P* = 0.542 FE). The predominant reason for early termination in the formula groups was the occurrence of an (S)AE (Control 19 subjects, Concept 25 subjects), others were lost to follow-up (Control 4 subjects, Concept 1 subject), nonspecified reason (Control 1 subject, Concept 1 subject), protocol violation (Control 1 subject), or withdrawal of informed consent (Control 1 subject). Demographic data were not apparently different between the IMF groups for the PP (**Table 2**) as well as ITT populations (**Supplemental Table 1**). It should be noted that the age at baseline seemed to be higher in the breastfed group compared with the IMF groups (median age at baseline in BF was 22 days compared with 5 days for IMF groups).

**TABLE 2:** Demographic characteristics of the per-protocol population^1^

	Control IMF (*n* = 83)	Concept IMF (*n* = 91)	Breastfed (*n* = 77)
Sex, *n* (%)			
Male	40 (48%)	44 (48%)	38 (49%)
Female	43 (52%)	47 (52%)	39 (51%)
Country, *n* (%)			
Belgium	44 (53%)	42 (46%)	30 (39%)
France	4 (5%)	6 (7%)	0 (0%)
Singapore	3 (4%)	3 (3%)	12 (16%)
Netherlands	32 (39%)	40 (44%)	35 (46%)
Age at baseline, *n* (%)			
≤14 d	64 (77%)	65 (71%)	26 (34%)
˃14 d	19 (23%)	26 (29%)	51 (66%)
Median, d	4 (3, 14)	5 (3, 15)	22 (4, 28)
Birth characteristics			
Weight, g	3360 (3040, 3560)	3335 (3085, 3568)	3370 (3140, 3635)
Length, cm	49 (48, 51)	50 (48, 51)	50 (49, 51)
Head circumference, cm	34 (34, 35.5)	34.5 (33.5, 35.5)	34.5 (33.5, 35.5)
Vaginal delivery, *n* (%)	63 (76%)	57 (63%)	57 (74%)
Caesarean section, *n* (%)	20 (24%)	34 (37%)	20 (26%)
Gestational age, wk	39.6 (38.4, 40.0)	39.6 (38.4, 40.1)	39.6 (38.7, 40.9)
Parental characteristics			
Maternal age, y	30 (24, 34)	31 (27, 35)	31 (29, 34)
Maternal university education, yes, *n* (%)	33 (40%)	33 (36%)	50 (65%)
Maternal BMI, kg/m^2^	23.6 (21.2, 25.6)	23.4 (21.5, 29.1)	23.6 (21.0, 27.1)
Paternal BMI, kg/m^2^	25.6 (23.2, 27.8)	24.8 (23.1, 27.8)	24.8 (23.4, 27.4)

1 Values are *n* (%) or median (IQR). IMF, infant milk formula.

### Growth outcomes

Equivalence in daily weight gain (g/d) from enrollment to 17 wk of age was demonstrated for the Concept compared with Control IMF (mean ± SD of 28.3 ± 4.8 compared with 29.4 ± 6.0 g/d) in the full PP population (difference in estimated means of −1.37 g/d; 90% CI: −2.71, −0.02 g/d), for infants aged ≤14 d at enrollment only (−1.09 g/d; 90% CI: −2.67, 0.49 g/d), as well as in the ITT population (−0.63 g/d; 90% CI: −1.83, 0.56 g/d). Compared with the breastfed reference group (daily weight gain of 26.8 ± 6.5 g/d), equivalence in daily weight gain (PP population) was demonstrated for the Concept IMF (difference in estimated means of 0.38 g/d; 90% CI: −0.99, 1.75 g/d; PC) and also suggested equivalence for the Control IMF, because the upper confidence limit coincided with the upper equivalence margin (difference in estimated means of 1.67 g/d; 90% CI: 0.19, 3.15 g/d; PC).

Mean weight, length, and head circumference values were highly similar for formula-fed as well as breastfed groups throughout the intervention period (**Table 3**; **Supplemental Table 2**). Equivalence in daily length and head circumference gain was demonstrated for IMF groups in the PP (irrespective of inclusion age) and ITT populations (data not shown). Compared with the breastfed reference, equivalence in length gain during the study was not demonstrated for the Concept IMF due to a higher length gain in this group, but was demonstrated for the Control IMF (data not shown). Head circumference gain of both IMF groups was not equivalent to the breastfed reference group (data not shown) due to higher gains in the formula groups.

**TABLE 3:** Anthropometric measures during the intervention period of the per-protocol population^1^

Measure	Postnatal age, wk	Control IMF (*n* = 83)	Concept IMF (*n* = 91)	Breastfed reference (*n* = 77)	*P* value Control vs Concept IMF
Weight, g	Baseline	3384 ± 456 (83)	3418 ± 475 (91)	3880 ± 667 (77)	0.938
	5	4303 ± 444 (74)	4341 ± 449 (75)	4409 ± 453 (35)	0.916
	8	4987 ± 507 (79)	4986 ± 493 (83)	5225 ± 551 (74)	0.892
	13	5935 ± 596 (71)	5879 ± 614 (77)	6063 ± 685 (70)	0.415
	17	6675 ± 683 (58)	6530 ± 661 (70)	6641 ± 795 (65)	0.157
Length, cm	Baseline	50.9 ± 2.2 (83)	50.9 ± 2.4 (91)	52.5 ± 2.8 (77)	0.995
	5	54.0 ± 1.8 (74)	54.3 ± 1.8 (75)	54.4 ± 1.8 (35)	0.471
	8	56.7 ± 2.0 (79)	57.0 ± 2.0 (83)	57.2 ± 1.8 (74)	0.287
	13	60.3 ± 2.3 (71)	60.6 ± 2.1 (77)	60.8 ± 2.0 (69)	0.244
	17	62.9 ± 2.4 (58)	62.9 ± 2.1 (70)	63.2 ± 2.1 (65)	0.404
Head circumference, cm	Baseline	35.1 ± 1.3 (83)	35.2 ± 1.3 (91)	36.1 ± 1.5 (77)	0.968
	5	37.4 ± 1.1 (74)	37.5 ± 1.0 (75)	37.3 ± 1.1 (34)	0.909
	8	38.7 ± 1.1 (79)	38.7 ± 1.1 (83)	38.7 ± 1.0 (74)	0.914
	13	40.2 ± 1.2 (71)	40.1 ± 1.1 (77)	40.1 ± 1.1 (70)	0.890
	17	41.5 ± 1.3 (58)	41.2 ± 1.2 (70)	41.1 ± 1.1 (65)	0.645

1 The data are presented as means ± SD (*n*). Differences in measures between groups were evaluated with the use of a parametric curves mixed model with the stratification factors as a fixed effect, and each subject's intercept and slope as random effects. *P < 0.05 for Concept compared with Control; **P < 0.01 for Concept compared with Control; no statistical testing compared to breastfeeding was done. IMF, infant milk formula.

**FIGURE 2:**
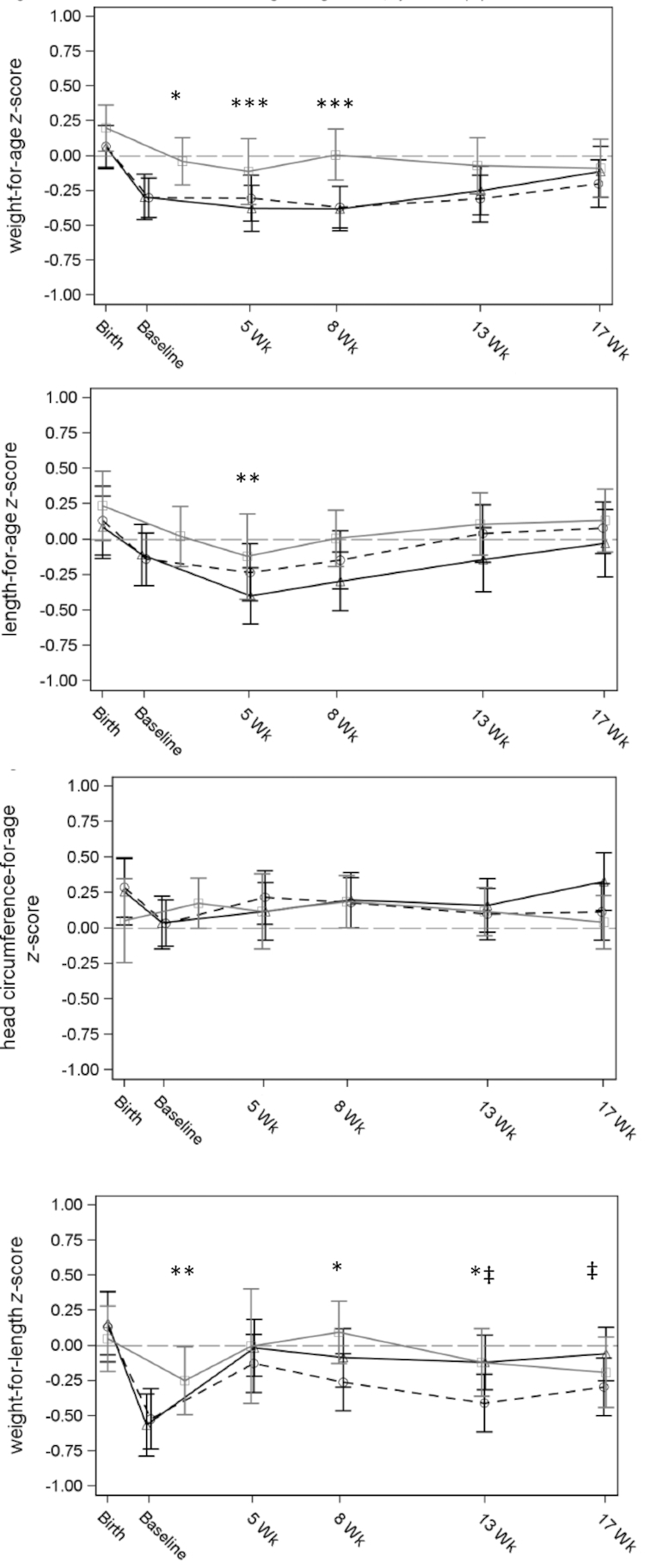
Mean (± 95% CI) weight-for-age, length-for-age (left and right upper panel), head-circumference-for-age, and weight-for-length (left and right lower panel) WHO growth standard *z* scores for the Control IMF (Δ, black solid line), Concept IMF (○, dashed black line), and the breastfed reference group (□, gray line) of the PP population. ‡Statistical difference between Concept and Control IMF (*P* < 0.05); *statistical difference between Concept IMF and breastfed group (*P* < 0.05); **statistical difference between Control IMF and breastfed group (*P* < 0.05); ***statistical difference between both IMF groups compared with breastfed group (*P* < 0.05). IMF, infant milk formula.

The mean weight-for-age, length-for-age, weight-for-length, and head circumference-for-age WHO *z* score values of both IMF groups were within a ±0.5 *z* score bandwidth (**Figure 2**), indicative for adequate growth. No significant differences in *z* scores were observed between IMF groups, apart from a slightly lower weight-for-length *z* score in the Concept compared with Control IMF group at 13 and 17 wk of age (Figure 2). The infants of the breastfed reference group had a numerically higher weight and length at birth compared with both IMF groups (Table 2), a difference that persisted throughout the intervention period as indicated by the higher *z* scores (Figure 2; nonsignificant after 8 wk of age). Additionally, a slightly higher weight-for-length *z* score was observed at 8 and 13 wk of age compared with the Concept IMF group in the PP (but not ITT) population (*P* < 0.05).

### Study product intake

The diaries to record formula intake were properly applied, with >90% of the parents completing the full 7-d diary preceding the visits. Infants in both IMF groups consumed an increasing amount of formula (mL/d) during the study period (**Table 4**). The median daily formula intake (mL/d) was lower in infants consuming the Concept IMF compared with Control IMF at 13 and 17 wk of age (*P* = 0.033 and *P* = 0.037 MW). However, daily formula intake per kg body weight at these time points did not show any statistically significant differences between Concept and Control IMF (*P* = 0.091 MW and *P = *0.181 MW at 13 and 17 wk of age).

**TABLE 4:** Average daily product intake during the intervention period of the per-protocol population^1^

Product intake	Postnatal age, wk	Control IMF (*n* = 83)	Concept IMF (*n* = 91)	*P* value^2^
Average daily intake,^3^ mL/d	5	719.6 (663.6, 788.6) (74)	714.3 (652.9, 782.9) (74)	0.607
	8	780.4 (710.0, 842.1) (78)	750.7 (690.0, 818.6) (83)	0.113
	13	837.1 (766.7, 947.1) (71)	790.0 (745.0, 877.7) (77)	0.033
	17	878.6 (824.3, 958.6) (58)	855.7 (780.0, 910.0) (69)	0.037
Average daily intake per kg body weight, mL · kg^–1^ · d^–1^	5	168.7 (152.3, 185.3) (74)	165.8 (155.0, 181.4) (74)	0.360
	8	156.1 (146.1, 168.0) (78)	151.0 (136.8, 164.3) (83)	0.071
	13	144.0 (130.6, 154.7) (71)	138.4 (128.1, 148.5) (77)	0.091
	17	133.7 (124.1, 143.6) (58)	128.8 (120.1, 137.2) (69)	0.181

1 The data are presented as median and interquartile range (*n*). IMF, infant milk formula.

2 The data were analyzed with the use of a pairwise comparison by Wilcoxon rank-sum tests.

3 At each time point the vast majority of parents (>90%) completed the full diary (7 d of formula intake recorded).

### Gastrointestinal tolerance

Daily stool frequency remained stable during the intervention period, with a slightly higher mean ± SD stool frequency in the Concept IMF (1.32 ± 0.69 stools/d) compared with the Control IMF group (1.11 ± 0.57 stools/d; *P* = 0.032; TT; **Table 5**) at 13 wk of age. Infants in the breastfed group showed a substantially higher daily stool frequency, although reducing over time. During the intervention period, most infants in the Concept and Control IMF groups had a stool consistency categorized as “soft stool.” In the Concept IMF group, the percentage of subjects with watery stools was higher compared with the Control IMF group at 5, 8, and 13 wk of age (23% compared with 1%, *P *< 0.001 FE; 28% compared with 5%, *P *< 0.001 FE; and 28% compared with 11%, *P* = 0.002 FE, respectively) with the Concept IMF group close to the consistency scores seen in the breastfed group (ranging from 21% to 31% of subjects with watery stools and from 69% to 79% with soft stools during 5–13 wk of age).

**TABLE 5:** Tolerance parameters in the Control IMF and Concept IMF groups, and the breastfed reference group^1^

Parameter	Age, wk	Severity	Control IMF (*n* = 108)	Concept IMF (*n* = 115)	Breastfed (*n* = 88)
Stool frequency (*n*/d)^2^	5	—	1.59 ± 1.13 (82)	1.64 ± 1.07 (82)	4.39 ± 1.74 (34)
	8	—	1.22 ± 0.92 (88)	1.38 ± 0.89 (93)	3.23 ± 2.14 (72)
	13	—	1.11 ± 0.57 (85)	1.32 ± 0.69 (89)*	2.29 ± 1.66 (69)
	17	—	1.35 ± 0.78 (81)	1.46 ± 0.79 (86)	1.95 ± 1.36 (67)
Diarrhea incidence^3^	5	—	3 (3.7%) (82)	11 (13.4%) (82)*	12 (35.3%) (34)
	8	—	2 (2.3%) (88)	13 (14.0%) (93)**	22 (30.6%) (72)
	13	—	3 (3.5%) (85)	13 (14.6%) (89)*	19 (27.5%) (69)
	17	—	7 (8.6%) (81)	10 (11.6%) (86)	13 (19.4%) (67)
Cramps^4^	5	Absent	27 (32.9%)	28 (34.1%)	10 (29.4%)
		Mild	42 (51.2%)	31 (37.8%)	18 (52.9%)
		Moderate	12 (14.6%)	20 (24.4%)	6 (17.6%)
		Severe	1 (1.2%)	3 (3.7%)	0 (0.0%)
	8	Absent	49 (55.7%)	38 (40.9%)	30 (41.7%)
		Mild	25 (28.4%)	37 (39.8%)	32 (44.4%)
		Moderate	13 (14.8%)	15 (16.1%)	7 (9.7%)
		Severe	1 (1.1%)	3 (3.2%)	3 (4.2%)
	13	Absent	58 (69.0%)	62 (69.7%)	43 (62.3%)
		Mild	22 (26.2%)	25 (28.1%)	22 (31.9%)
		Moderate	3 (3.6%)	2 (2.2%)	4 (5.8%)
		Severe	1 (1.2%)	0 (0.0%)	0 (0.0%)
	17	Absent	67 (83.8%)	65 (75.6%)	54 (80.6%)
		Mild	12 (15.0%)	19 (22.1%)	12 (17.9%)
		Moderate	1 (1.3%)	2 (2.3%)	1 (1.5%)
		Severe	0 (0.0%)	0 (0.0%)	0 (0.0%)
Regurgitation^4^	5‡	Absent	32 (39.0%)	18 (22.0%)	7 (20.6%)
		Mild	41 (50.0%)	47 (57.3%)	22 (64.7%)
		Moderate	8 (9.8%)	17 (20.7%)	5 (14.7%)
		Severe	1 (1.2%)	0 (0.0%)	0 (0.0%)
	8	Absent	36 (41.4%)	31 (33.7%)	22 (31.0%)
		Mild	42 (48.3%)	48 (52.2%)	41 (57.7%)
		Moderate	7 (8.0%)	10 (10.9%)	6 (8.5%)
		Severe	2 (2.3%)	3 (3.3%)	2 (2.8%)
	13^‡^	Absent	41 (48.8%)	24 (27.3%)	30 (43.5%)
		Mild	34 (40.5%)	49 (55.7%)	34 (49.3%)
		Moderate	9 (10.7%)	14 (15.9%)	4 (5.8%)
		Severe	0 (0.0%)	1 (1.1%)	1 (1.4%)
	17^‡^	Absent	41 (51.3%)	26 (30.6%)	29 (43.3%)
		Mild	32 (40.0%)	53 (62.4%)	33 (49.3%)
		Moderate	7 (8.8%)	5 (5.9%)	5 (7.5%)
		Severe	0 (0.0%)	1 (1.2%)	0 (0.0%)

1 Values are means ± SD or prevalence (%) and number of infants (*n*) of the ITT population, and n (%) for severity results of cramps and regurgitation. IMF, infant milk formula; ITT, intention-to-treat.

2 Stool frequency comparisons were tested through the use of a *t* test.

3 Applying the WHO definition of having ≥3 watery stools on 1 day; incidence scores were tested through the use of a chi-square test.

4 Severity results are placed into a severity category based on each subject's mean score of the 7-d diary prior to the visit rounded to an integer number. Severity score comparisons were tested through the use of Fisher's exact test. *P < 0.05 for Concept compared with Control; **P < 0.01 for Concept compared with Control; ^‡^*P* < 0.05 for distribution of subjects across severity categories for Concept compared with Control; no statistical testing compared with breastfeeding was done.

The percentage of infants consuming Concept or Control IMF was not apparently different across cramps severity categories (absent, mild, moderate, or severe) and was comparable to the breastfed reference group at all time points (Table 5). A total of 38% and 51% of the infants consuming Concept or Control IMF, respectively, experienced “mild” cramps at 5 wk of age, but that reduced over time, being absent in most formula-fed infants at the end of the intervention period. The clear majority of formula-fed subjects experienced no or mild regurgitation throughout the intervention period, with a higher percentage of subjects with mild to moderate regurgitation in the Concept IMF compared to the Control IMF group at 5, 13, and 17 wk of age (*P *= 0.024, *P* = 0.017, and *P* = 0.013 FE), with values comparable to the breastfed group. Most formula-fed infants (>70% and >85% at all time points) did not experience any vomiting or diaper rash, respectively, without any statistical significant differences between IMF groups and with comparable values to the breastfed group (data not shown).

### Adverse events

The safety analysis was performed on the AST population (112 subjects in the Concept IMF group and 103 subjects in the Control IMF group). Overall, 21 SAEs were reported for 19 randomly assigned subjects (8.8%) during the intervention period. For comparison, in the breastfed reference group, 5 SAEs were reported in 3 infants (3.4%) during the study period. The percentage of subjects with ≥1 SAEs overall as well as by severity score was not statistically significant different between both formula groups. Two SAEs—a case of gastroenteritis and an infant with cow's milk intolerance—in 2 subjects of the Control IMF group were reported to be possibly related to the study product. No statistically significant differences were observed in the percentage of subjects with AEs overall, or with AEs by severity, apart from a lower percentage of subjects with the AE (preferred term) dry skin in the Concept IMF compared with the Control IMF group [1 event in 1 (0.9%) subject compared with 9 events in 7 (6.8%) subjects; *P* = 0.030 FE]. During the study, dry skin was never reported as an AE for the breastfed reference group. In the formula-fed infants, the most commonly observed AEs were infections [with 155 events in 95 formula-fed subjects (44.2%) and 36 events in 24 breastfed subjects (27.3%)] and GI disorders [235 events in 122 formula-fed subjects (56.7%) and 41 events in 26 breastfed subjects (29.5%)]. During the intervention period, the occurrence of diarrhea was higher in the Concept IMF compared with the Control IMF group [16 events in 15 subjects (13.4%) compared with 8 events in 8 subjects (7.8%); *P* = 0.194 FE]. For comparison, in the breastfed reference group, 3 events in 2 infants were reported (2.3%). Although the difference in reported diarrhea was not statistically significant between intervention groups, a more detailed evaluation was performed. The mean duration of diarrhea (11.7 d for Concept IMF and 20.4 d for Control IMF) was not statistically significant different between study groups, and most events occurred ˃57 d after start of study product intake, indicating a proper tolerance in the first weeks of use. A second detailed evaluation was performed for vomiting due to a higher, not statistically significant occurrence of vomiting in the Concept IMF compared with the Control IMF group [30 events in 20 (17.9%) subjects compared with 18 events in 14 (13.6%) subjects; *P* = 0.456 FE]. For comparison, 9 events in 7 subjects were reported in the breastfed group (8%). Most subjects in the Control IMF group had an onset of vomiting between 1 and 28 d after the start of the study product intake, whereas in the Concept IMF group the timing was between 15 and 56 d after the start of the study product intake. Product relatedness scores of the vomiting events were not apparently different between both IMF groups. The 2 detailed evaluations described are performed as standard procedure for clinical evaluation and serve as a precaution to identify any adverse impact of the intervention products. Based on these assessments and described AE outcomes, there was no safety concern related to the occurrence of any (S)AEs during the study.

### Plasma vitamins

At week 13, a voluntary capillary blood sample was obtained from 51 infants consuming Concept IMF (44%), 40 infants consuming Control IMF (37%), and 38 breastfed infants (43%). The mean ± SD plasma vitamin A concentration of infants in the Concept IMF group was not different from those in the Control IMF group (1.68 ± 0.32 compared with 1.61 ± 0.39 μmol/L; *P* = 0.423 TT). A mean ± SD concentration of 1.21 ± 0.36 μmol/L was observed in the breastfed infants. Most infants [43 (88%) for Concept IMF and 27 (82%) for Control IMF] had plasma values of vitamin A within the adequate range (0.7 μmol/L ≤ value ≤ 2 μmol/L). Mean ± SD vitamin E levels were not apparently different in infants of both IMF groups (32.29 ± 6.27 μmol/L for Concept IMF and 32.73 ± 6.46 μmol/L for Control IMF; *P* = 0.62 TT) and all subjects of both formula-fed and breastfed groups had values within the adequate range (11–50 μmol/L).

## Discussion

This double-blind, randomized, controlled trial evaluated a concept IMF with lipid droplets that are large, milk phospholipid–coated, and contain dairy lipids (48%), features that are inspired by the large, triple-layer membrane–enveloped lipid droplets in human milk (12). We observed an equivalent daily weight gain for infants fed the Concept IMF compared with those fed a standard IMF up to 17 wk of age despite a slightly lower formula intake at 13 and 17 wk of age in the Concept IMF group compared with the standard IMF group. The Concept IMF supported adequate growth, was well tolerated, and no major safety concerns were revealed as reflected by the absence of clinically relevant differences in number, severity, relatedness, or type of (S)AEs, and in plasma vitamin A and E levels.

Previously, a noninferiority trial showed that enrichment of standard formula with milk phospholipid fractions in comparable quantities to those used in the current study (45 or 65 mg/100 mL compared with 55 mg/100 mL, respectively) was well tolerated and resulted in a noninferior daily infant weight gain (0–4 mo of age) (24). In addition, no significant differences in length or head circumference gains were observed, and weight-for-age, length-for-age, and head-circumference-for-age *z* scores were within ±1 SD margins, which was deemed to be indicative of adequate growth (24). In a randomized, controlled efficacy trial, the impact of consuming a low-protein, low-energy, milk phospholipid (70 mg/100 mL)–supplemented experimental formula from <2 to 6 mo of age compared with standard formula on infant growth was evaluated (25). No significant differences in growth patterns were observed, including weight-for-age, length-for-age, head-circumference-for-age, or BMI-for-age *z* scores, as well as body fat percentage at 4 mo, between formula groups, although the infants consuming the experimental formula did compensate for the reduced energy and protein densities by increasing their volume intake (25). In the current study, the milk phospholipids are present in the Concept IMF as an emulsifier of the large lipid droplets rather than as a mere ingredient. However, the Concept IMF, containing the milk phospholipids and in addition also dairy lipids, likewise supports an adequate growth, evident from the demonstrated equivalent daily weight gain, length gain, and head circumference gain compared with those fed standard formula, irrespective of age at inclusion (≤14 d of age or ≤35 d of age). In contrast to the previous study, formula intake was slightly lower in the Concept IMF group compared with the Control IMF group. Infant growth outcomes in both IMF groups were close (equivalent) to those of breastfed infants in the current study as well as to the WHO growth standards for weight, length, and head circumference, indicating normal growth.

The prebiotic mixture scGOS/lcFOS (9:1) present in the Concept and Control IMF has been shown to stimulate the intestinal colonization with bifidobacteria, resulting in beneficial effects on immune function, and has a stool-softening effect (26–29). As expected, the stool consistency of most formula-fed infants in the current study was categorized as “soft.” Some differences in stool consistency scores were observed between formula groups, notably the (near) absence of subjects with hard stools and an increased percentage of subjects with watery stools in the Concept IMF compared with the Control IMF group, closely mirroring the stool consistency of breastfed infants. Timby et al. (30) showed that mere milk phospholipid enrichment of formula did not significantly affect stool consistency outcomes in infants, and this remained distinctly different from that of breastfed infants. However, others have demonstrated a significant stool-softening effect of increased levels of sn-2 palmitate (39–66% of total palmitic acid) in formula milks, either or with or without prebiotics, resulting in more “watery” and less “hard or formed” stools (8–11, 31). Hence, it is likely that in the present study the introduction of dairy lipids, rich in sn-2 palmitate, explains the observed stool-softening effects of the Concept IMF.

Because lipid droplet size and coating has been shown to affect lipid digestion (32), it is possible that the bioavailability of fat-soluble vitamins in the Concept IMF could be affected. However, no differences in plasma levels of vitamins A and E were detected between infants in the Control IMF and Concept IMF groups, suggesting similarity in bioavailability of these fat-soluble vitamins. Our findings are in line with those of Borel et al. (33) who found no differences in the efficiency of intestinal absorption of vitamins A and E between formulas with lipid droplet sizes of 0.7 and 10 μm in healthy adults.

Overall, the reported types of (S)AEs are typical for young infants, and no clinically relevant difference in the frequency, relatedness, type, or severity of (S)AEs was observed between formula groups. The drop-out rate (25%) and reasons for drop-out were similar between the Concept IMF and Control IMF groups, with the main reason being the occurrence of an AE. Furthermore, none of the parameters we assessed to evaluate tolerability indicated any AEs of the Concept IMF. This evaluation included a detailed investigation on the observed significantly higher occurrence, although numerically low and close to breastfed reference values, of diarrhea and vomiting in the Concept IMF group. Interestingly, the prevalence of watery stools was higher in the breastfed reference group, but the occurrence of reported diarrhea as an AE was lower than in the Concept IMF group (6.8% compared with 15.2% of infants with at least 1 occasion of diarrhea reported as an AE). We assume this can be attributed to a greater parental, and possibly also pediatrician, concern over watery stools in infants fed an intervention formula compared with breastfed infants (10).

Finally, some limitations of this study need to be addressed. Compared with the Control IMF, the lipid moiety of the Concept IMF exhibited the following differences: *1*) an altered lipid droplet structure, *2*) milk phospholipids (as emulsifier), and *3*) a 3-fold increased sn-2 palmitic acid concentration (dairy lipids). This implies that differences in study outcomes cannot be attributed to the single effect of either the lipid composition or lipid structure separately. This study was conducted at 17 study sites. Although all investigators were well informed and received training or strict manuals for the measurement and sampling procedures, this may have increased variation in study outcomes. Importantly, however, the blinded interim analysis confirmed that the intended population size was sufficient to substantiate the primary equivalence analysis outcomes. In addition to site-specific variation, the inclusion of both Caucasian and Asian infants may have introduced additional variation, as a consequence of potential differences in growth trajectories (34). However, the number of Asian infants included in the study was small, and, as a precaution, region was included as a stratification factor. Blood samples were collected on a voluntary basis and were therefore only available for a subgroup of infants. Given the fact that the baseline characteristics of this subgroup did not show apparent differences from the total population recruited, it is anticipated that this selection has introduced only a limited degree of bias and is representative for total population outcomes.

In conclusion, a concept IMF with large, milk phospholipid–coated lipid droplets containing dairy lipids supports adequate growth and is well tolerated and safe for use in infants. It is anticipated that this new dimension of lipid quality could also bring the functional properties of IMFs closer to that observed in breastfed infants. Preclinical evidence shows that early life exposure to the concept IMF leads to an improved body composition and better metabolic and cognitive outcomes in adult mice. Future longitudinal clinical studies are required to evaluate the potential long-term programming effect of lipid droplet characteristics in infant nutrition on growth trajectories, body composition, and metabolic development, as well as other health outcomes.

## Supplementary Material

nqy322_Supplemental_File

## Notes

This study was sponsored by Nutricia Research, which provided financial support to the participating centers to conduct the study. MA-B, SS, and DA are employees of Nutricia Research.

Supplemental Tables 1 and 2 is available from the “Supplementary data” link in the online posting of the article and from the same link in the online table of contents at https://academic.oup.com/ajcn/.

Abbreviations used: AE, adverse event; AST, all subjects treated; C, chi-square test; FE, Fisher's exact test; GI, gastrointestinal; IMF, infant milk formula; ITT, intention-to-treat; MW, Mann-Whitney *U* test; PP, per-protocol; PC, parametric growth curve; SAE, serious adverse event; scGOS/lcFOS, a specific prebiotic mixture consisting of short-chain galacto-oligosaccharides and long-chain fructo-oligosaccharides in a ratio of 9:1; TT, 2-sample *t* test.

## References

[1] Jensen RG Lipids in human milk. Lipids. 1999;34(12):1243–71. 10652985 10.1007/s11745-999-0477-2

[2] Gallier S, Gragson D, Jimenez-Flores R, Everett D Using confocal laser scanning microscopy to probe the milk fat globule membrane and associated proteins. J Agric Food Chem. 2010;58(7):4250–7. 20218614 10.1021/jf9032409 PMC2853928

[3] Lopez C, Menard O Human milk fat globules: polar lipid composition and in situ structural investigations revealing the heterogeneous distribution of proteins and the lateral segregation of sphingomyelin in the biological membrane. Colloids and Surf B Biointerfaces. 2011;83(1):29–41. 21126862 10.1016/j.colsurfb.2010.10.039

[4] Michalski MC, Briard V, Michel F, Tasson F, Poulain P Size distribution of fat globules in human colostrum, breast milk, and infant formula. J Dairy Sci. 2005;88(6):1927–40. 15905422 10.3168/jds.S0022-0302(05)72868-X

[5] Zou L, Pande G, Akoh CC Infant formula fat analogs and human milk fat: new focus on infant developmental needs. Annu Rev Food Sci Technol. 2016;7:139–65. 26934172 10.1146/annurev-food-041715-033120

[6] Lien EL, Boyle FG, Yuhas R, Tomarelli RM, Quinlan P The effect of triglyceride positional distribution on fatty acid absorption in rats. J Pediatr Gastroenterol Nutr. 1997;25(2):167–74. 9252903 10.1097/00005176-199708000-00007

[7] Tomarelli RM, Meyer BJ, Weaber JR, Bernhart FW Effect of positional distribution on the absorption of the fatty acids of human milk and infant formulas. J Nutr. 1968;95(4):583–90. 5665659 10.1093/jn/95.4.583

[8] Nowacki J, Lee HC, Lien R, Cheng SW, Li ST, Yao M, Northington R, Jan I, Mutungi G Stool fatty acid soaps, stool consistency and gastrointestinal tolerance in term infants fed infant formulas containing high sn-2 palmitate with or without oligofructose: a double-blind, randomized clinical trial. Nutr J. 2014;13:105. 25373935 10.1186/1475-2891-13-105 PMC4273321

[9] Yao M, Lien EL, Capeding MR, Fitzgerald M, Ramanujam K, Yuhas R, Northington R, Lebumfacil J, Wang L, DeRusso PA Effects of term infant formulas containing high sn-2 palmitate with and without oligofructose on stool composition, stool characteristics, and bifidogenicity. J Pediatr Gastroenterol Nutr. 2014;59(4):440–8. 24840511 10.1097/MPG.0000000000000443 PMC4222706

[10] Kennedy K, Fewtrell MS, Morley R, Abbott R, Quinlan PT, Wells JC, Bindels JG, Lucas A Double-blind, randomized trial of a synthetic triacylglycerol in formula-fed term infants: effects on stool biochemistry, stool characteristics, and bone mineralization. Am J Clin Nutr. 1999;70(5):920–7. 10539755 10.1093/ajcn/70.5.920

[11] Bar-Yoseph F, Lifshitz Y, Cohen T, Malard P, Xu C SN2-palmitate reduces fatty acid excretion in Chinese formula-fed infants. J Pediatr Gastroenterol Nutr. 2016;62(2):341–7. 26334255 10.1097/MPG.0000000000000971 PMC4732008

[12] Gallier S, Vocking K, Post JA, Van De Heijning B, Acton D, Van Der Beek EM, van Baalen T A novel infant milk formula concept: Mimicking the human milk fat globule structure. Colloids Surf B Biointerfaces. 2015;136:329–39. 26432620 10.1016/j.colsurfb.2015.09.024

[13] Van den Braak C, Thomassen G, Acton D, Ludwig T, Abrahamse E A concept infant formula with large, phospholipid coated droplets demonstrates slow in vitro gastric lipolysis as compared to regular infant formula. J Pediatr Gastroenterol Nutr. 2015;60(S1):740 (abstract).

[14] Baumgartner S, van de Heijning BJM, Acton D, Mensink RP Infant milk fat droplet size and coating affect postprandial responses in healthy adult men: a proof-of-concept study. Eur J Clin Nutr. 2017;71(9):1108–13. 28422122 10.1038/ejcn.2017.50

[15] Oosting A, van Vlies N, Kegler D, Schipper L, Abrahamse-Berkeveld M, Ringler S, Verkade HJ, van der Beek EM Effect of dietary lipid structure in early postnatal life on mouse adipose tissue development and function in adulthood. Br J Nutr. 2014;111(2):215–26. 23845308 10.1017/S0007114513002201

[16] Baars A, Oosting A, Engels E, Kegler D, Kodde A, Schipper L, Verkade HJ, van der Beek EM Milk fat globule membrane coating of large lipid droplets in the diet of young mice prevents body fat accumulation in adulthood. Br J Nutr. 2016;115(11):1930–7. 27040581 10.1017/S0007114516001082 PMC4863696

[17] Schipper L, van Dijk G, Broersen LM, Loos M, Bartke N, Scheurink AJ, van der Beek EM A postnatal diet containing phospholipids, processed to yield large, phospholipid-coated lipid droplets, affects specific cognitive behaviors in healthy male mice. J Nutr. 2016;146(6):1155–61. 27146919 10.3945/jn.115.224998

[18] Food and Drug Administration. Guidance for industry: demonstration of the quality factor requirements under 21 CFR 106.96(i) for eligible infant formulas. 2014 [Internet]. https://www.fda.gov/food/guidanceregulation/guidancedocumentsregulatoryinformation/ucm400036.htm[cited 26 February, 2018].

[19] EFSA Panel on Dietetic Products NaAN. Scientific opinion on the essential composition of infant and follow-on formulae. EFSA J. 2014;12(7):3760.

[20] Visser GH, Eilers PH, Elferink-Stinkens PM, Merkus HM, Wit JM New Dutch reference curves for birthweight by gestational age. Early Hum Dev. 2009;85(12):737–44. 19914013 10.1016/j.earlhumdev.2009.09.008

[21] World Health Organization Multicenter Growth Reference Study Group. WHO child growth standards based on length/height, weight and age. Acta Paediatr. 2006;Suppl 450:76–85. 10.1111/j.1651-2227.2006.tb02378.x 16817681

[22] Bekkali N, Hamers SL, Reitsma JB, Van Toledo L, Benninga MA Infant stool form scale: development and results. J Pediatr. 2009;154(4):521–6. 19054528 10.1016/j.jpeds.2008.10.010

[23] Drossman DA, Dumitrascu DL Rome III: new standard for functional gastrointestinal disorders. J Gastrointestin Liver Dis. 2006;15(3):237–41. 17013448

[24] Billeaud C, Puccio G, Saliba E, Guillois B, Vaysse C, Pecquet S, Steenhout P Safety and tolerance evaluation of milk fat globule membrane-enriched infant formulas: a randomized controlled multicenter non-inferiority trial in healthy term infants. Clin Med Insights Pediatr. 2014;8:51–60. 25452707 10.4137/CMPed.S16962 PMC4219856

[25] Timby N, Domellof E, Hernell O, Lonnerdal B, Domellof M Neurodevelopment, nutrition, and growth until 12 mo of age in infants fed a low-energy, low-protein formula supplemented with bovine milk fat globule membranes: a randomized controlled trial. Am J Clin Nutr. 2014;99(4):860–8. 24500150 10.3945/ajcn.113.064295

[26] Scholtens PA, Goossens DA, Staiano A Stool characteristics of infants receiving short-chain galacto-oligosaccharides and long-chain fructo-oligosaccharides: a review. World J Gastroenterol. 2014;20(37):13446–52. 25309075 10.3748/wjg.v20.i37.13446 PMC4188896

[27] Knol J, Scholtens P, Kafka C, Steenbakkers J, Gro S, Helm K, Klarczyk M, Schopfer H, Bockler HM, Wells J Colon microflora in infants fed formula with galacto- and fructo-oligosaccharides: more like breast-fed infants. J Pediatr Gastroenterol Nutr. 2005;40(1):36–42. 15625424 10.1097/00005176-200501000-00007

[28] Arslanoglu S, Moro GE, Schmitt J, Tandoi L, Rizzardi S, Boehm G Early dietary intervention with a mixture of prebiotic oligosaccharides reduces the incidence of allergic manifestations and infections during the first two years of life. J Nutr. 2008;138(6):1091–5. 18492839 10.1093/jn/138.6.1091

[29] Moro G, Arslanoglu S, Stahl B, Jelinek J, Wahn U, Boehm G A mixture of prebiotic oligosaccharides reduces the incidence of atopic dermatitis during the first six months of age. Arch Dis Child. 2006;91(10):814–9. 16873437 10.1136/adc.2006.098251 PMC2066015

[30] Timby N, Hernell O, Vaarala O, Melin M, Lonnerdal B, Domellof M Infections in infants fed formula supplemented with bovine milk fat globule membranes. J Pediatr Gastroenterol Nutr. 2015;60(3):384–9. 25714582 10.1097/MPG.0000000000000624

[31] Carnielli VP, Luijendijk IH, Van Goudoever JB, Sulkers EJ, Boerlage AA, Degenhart HJ, Sauer PJ Structural position and amount of palmitic acid in infant formulas: effects on fat, fatty acid, and mineral balance. J Pediatr Gastroenterol Nutr. 1996;23(5):553–60. 8985844 10.1097/00005176-199612000-00007

[32] Armand M, Pasquier B, Andre M, Borel P, Senft M, Peyrot J, Salducci J, Portugal H, Jaussan V, Lairon D Digestion and absorption of 2 fat emulsions with different droplet sizes in the human digestive tract. Am J Clin Nutr. 1999;70(6):1096–106. 10584056 10.1093/ajcn/70.6.1096

[33] Borel P, Pasquier B, Armand M, Tyssandier V, Grolier P, Alexandre-Gouabau MC, Andre M, Senft M, Peyrot J, Jausan V et al. Processing of vitamin A and E in the human gastrointestinal tract. Am J Physiol Gastrointest Liver Physiol. 2001;280(1):G95–G103. 11123202 10.1152/ajpgi.2001.280.1.G95

[34] Muhardi L, Abrahamse-Berkeveld M, Acton D, van der Beek EM Differences in the anthropometry of Asian children and its role in metabolic health in later life: A narrative review. Obes Res Clin Pract. 2016;10 Suppl 1:S3–S16. 27389317 10.1016/j.orcp.2016.04.002

