# Supplementary material for: An infant formula with large, milk phospholipid–coated lipid droplets containing a mixture of dairy and vegetable lipids supports adequate growth and is well tolerated in healthy, term infants
Source: Am J Clin Nutr. 2019 Feb 22;109(3):586–96. doi: 10.1093/ajcn/nqy322 (PMC6408203; doi:10.1093/ajcn/nqy322)
Supplement: nqy322_Supplemental_File [file nqy322_supplemental_file.docx]

Supplemental Table 1. Demographic characteristics of the intention to treat population

|  | Statistic | Control IMF^1^  (N = 108) | Concept IMF  (N = 115) | Breastfed  (N = 88) |
| --- | --- | --- | --- | --- |
| *Sex* |  |  |  |  |
| Male | n (%) | 53 (49%) | 55 (48%) | 45 (51%) |
| Female | n (%) | 55 (51%) | 60 (52%) | 43 (49%) |
| *Country* |  |  |  |  |
| Belgium | n (%) | 64 (57%) | 56 (49%) | 39 (44%) |
| France | n (%) | 5 (5%) | 8 (7%) | 0 (0%) |
| Singapore | n (%) | 4 (4%) | 5 (4%) | 12 (14%) |
| Netherlands | n (%) | 37 (34%) | 46 (40%) | 37 (42%) |
| *Age at baseline (d)* |  |  |  |  |
| Age ≤ 14 days | n (%) | 83 (77%) | 86 (75%) | 34 (39%) |
| Age ˃ 14 days | n (%) | 25 (23%) | 29 (25%) | 54 (61%) |
| Median age (d) | Median (IQR) | 4 (3-14) | 5 (3-15) | 20 (4-28) |
| *Birth characteristics* |  |  |  |  |
| Weight (g) | Median (IQR) | 3310 (3005-3555) | 3350 (3080 – 3620) | 3370 (3135-3612) |
| Length(cm) | Median (IQR) | 49 (48-51) | 50 (48-51) | 50 (49-51) |
| Head circumference (cm) | Median (IQR) | 34 (33.5-35.5) | 34.5 (33.5-35.5) | 34.5 (33.0-35.5) |
| Vaginal delivery | n (%) | 85 (79%) | 76 (66%) | 65 (74%) |
| Caesarean section | n (%) | 23 (21%) | 39 (34%) | 23 (26%) |
| Gestational age (wk) | Median (IQR) | 39.6 (38.4-40.0) | 39.4 (38.4-40.1) | 39.4 (38.4-40.9) |
| *Parental characteristics* |  |  |  |  |
| Maternal age (y) | Median (IQR) | 30 (27-34) | 30 (27-34) | 31 (28-34) |
| Maternal university education (yes) | n (%) | 44 (41%) | 45 (39%) | 57 (65%) |
| Maternal BMI (kg/m^2^) | Median (IQR) | 24 (21-26) | 23 (21-29) | 24 (21-28) |
| Paternal BMI (kg/m^2^) | Median (IQR) | 26 (23-27) | 25 (23 -28) | 25 (23-27) |

^1^IMF; infant milk formula

**Supplemental Table 2. Anthropometric measures during the intervention period of the intention to treat population^1^**

| Postnatal age | Control IMF^2^  (n = 108) | Concept IMF  (n = 91) | Breastfed reference (n = 88) | *P-value Control vs Concept IMF* |
| --- | --- | --- | --- | --- |
| *Weight (g)* |  |  |  |  |
| Baseline | 3366 ± 465 (107) | 3411 ± 470 (115) | 3828 ± 672 (88) | 0.374 |
| 5 weeks | 4275 ± 463 (81) | 4360 ± 460 (84) | 4450 ± 484 (38) | 0.284 |
| 8 weeks | 4946 ± 514 (89) | 5013 ± 486 (95) | 5224 ± 547 (75) | 0.366 |
| 13 weeks | 5892 ± 604 (88) | 5920 ± 605 (89) | 6056 ± 682 (71) | 0.752 |
| 17 weeks | 6598 ± 674 (81) | 6577 ± 665 (87) | 6639 ± 774 (69) | 0.800 |
| *Length (cm)* |  |  |  |  |
| Baseline | 50.8 ± 2.2 (107) | 50.9 ± 2.3 (115) | 52.4 ± 2.7 (88) | 0.649 |
| 5 weeks | 53.9 ± 1.9 (81) | 54.3 ± 1.9 (84) | 54.8 ± 1.8 (38) | 0.233 |
| 8 weeks | 56.6 ± 2.0 (89) | 56.9 ± 2.0 (95) | 57.2 ± 1.7 (75) | 0.138 |
| 13 weeks | 60.1 ± 2.3 (88) | 60.5 ± 2.2 (89) | 60.7 ± 2.1 (70) | 0.151 |
| 17 weeks | 62.7 ± 2.3 (81) | 63.0 ± 2.1 (87) | 63.1 ± 2.1 (69) | 0.345 |
| *Head circumference (cm)* |  |  |  |  |
| Baseline | 35.0 ± 1.4 (107) | 35.2 ± 1.3 (115) | 36.0 ± 1.5 (88) | 0.309 |
| 5 weeks | 37.3 ± 1.1 (81) | 37.5 ± 1.1 (84) | 37.4 ± 1.1 (37) | 0.148 |
| 8 weeks | 38.6 ± 1.1 (89) | 38.7 ± 1.1 (95) | 38.7 ± 1.0 (75) | 0.144 |
| 13 weeks | 40.1 ± 1.1 (88) | 40.2 ± 1.1 (89) | 40.1 ± 1.0 (71) | 0.309 |
| 17 weeks | 41.4 ± 1.2 (81) | 41.2 ± 1.2 (87) | 41.1 ± 1.1 (69) | 0.749 |

^1^The data are presented as means ± SD (n).^2^ IMF; infant milk formula. Differences in measures between groups were evaluated using Parametric curves mixed model with the stratification factors as a fixed effect, and each subject’s intercept and slope as random effects. *P<0.05 for Concept vs Control **P<0.01 for Concept vs. Control; No statistical testing compared to breastfeeding was done.
